# ACTG-HIV symptoms changes in patients switched to RPV/FTC/TDF due to previous intolerance to CART. Interim analysis of the PRO-STR study

**DOI:** 10.7448/IAS.17.4.19814

**Published:** 2014-11-02

**Authors:** Daniel Podzamczer, Nerea Rozas, Pere Domingo, Antonio Ocampo, Eva Van den Eynde, Elisabeth Deig, Antonio Vergara, Hernando Knobel, Juan Pasquau, Antonio Antela, Manuel Crespo, Bonaventure Clotet, Jessica Muñoz, Pedro Fernandez, Paloma Geijo, Eduardo Rodríguez de Castro, Julio Diz, Araceli Casado, Covadonga Torres

**Affiliations:** 1 Infectious Diseases, Hospital Universitari de Bellvitge, Barcelona, Spain; 2 Infectious Diseases, Hospital de la Santa Creu y Sant Pau, Barcelona, Spain; 3 Servicio de Medicina Interna, Hospital Xeral de Vigo, Pontevedra, Spain; 4 Servicio de Medicina Interna, Hospital General de Granollers, Granollers, Spain; 5 Servicio de Enfermedades Infecciosas, Hospital de Especialidades de Puerto Real, Cádiz, Spain; 6 Servicio de Enfermedades Infecciosas, Hospital del Mar, Barcelona, Spain; 7 Servicio de Enfermedades Infecciosas, Hospital Universitario Virgen de las Nieves, Granada, Spain; 8 Servicio de Enfermedades Infecciosas, Complejo Hospitalario Universitario de Santiago, A Coruña, Spain; 9 Servicio de Enfermedades Infecciosas, Hospital Vall d′ Hebrón, Barcelona, Spain; 10 Servicio de Enfermedades Infecciosas, Hospital Universitari Germans Trias i Pujol, Barcelona, Spain; 11 Servicio de Enfermedades Infecciosas, Hospital de la Santa Creu y Sant Pau, Barcelona, Spain; 12 Servicio de Enfermedades Infecciosas, Hospital Can Misses, Ibiza, Spain; 13 Servicio de Enfermedades Infecciosas, Hospital General Virgen de la Luz, Cuenca, Spain; 14 Servicio de Enfermedades Infecciosas, Hospital Mateu Orfila, Mahon, Spain; 15 Servicio de Enfermedades Infecciosas, Hospital Montecelo, A Coruña, Spain; 16 Pharmacoeconomics & Outcomes Research Iberia, Estadística, Madrid, Spain

## Abstract

**Introduction:**

Tolerability and convenience are crucial aspects for the long-term success of combined antiretroviral therapy (cART). The aim of this study was to investigate the impact in routine clinical practice of switching to the single tablet regimen (STR) RPV/FTC/TDF in patients with intolerance to previous cART, in terms of patients’ well-being, assessed by several validated measures.

**Methods:**

Prospective, multicenter study. Adult HIV-infected patients with viral load under 1.000 copies/mL while receiving a stable ART for at least the last three months and switched to RPV/FTC/TDF due to intolerance of previous regimen, were included. Analyses were performed by ITT. Presence/magnitude of symptoms (ACTG-HIV Symptom Index), quality of life (EQ-5D, EUROQoL & MOS-HIV), adherence (SMAQ), preference of treatment and perceived ease of medication (ESTAR) through 48 weeks were performed.

**Results:**

Interim analysis of 125 patients with 16 weeks of follow up was performed. 100 (80%) were male, mean age 46 years. Mean CD4 at baseline was 629.5±307.29 and 123 (98.4%) had viral load <50 copies/mL; 15% were HCV co-infected. Ninety two (73.6%) patients switched from a NNRTI (84.8% from EFV/FTC/TDF) and 33 (26.4%) from a PI/r. The most frequent reasons for switching were psychiatric disorders (51.2%), CNS adverse events (40.8%), gastrointestinal (19.2%) and metabolic disorders (19.2%). At the time of this analysis (week 16), four patients (3.2%) discontinued treatment: one due to adverse events, two virologic failures and one with no data. A total of 104 patients (83.2%) were virologically suppressed (<50 copies/mL). The average degree of discomfort in the ACTG-HIV Symptom Index significantly decreased from baseline (21±15.55) to week 4 (10.89±12.36) & week 16 (10.81±12.62), p<0.001. In all the patients, quality of life tools showed a significant benefit in well-being of the patients (Table 1). Adherence to therapy significantly and progressively increased (SMAQ) from baseline (54.4%) to week 4 (68%), p<0.001 and to week 16 (72.0%), p<0.001.

**Conclusions:**

Switching to RPV/FTC/TDF from another ARV regimen due to toxicity, significantly improved the quality of life of HIV-infected patients, both in mental and physical components, and improved adherence to therapy while maintaining a good immune and virological response.

**Table 1:** Changes in quality of life tests alter switching to RPV/FTC/TDF

	(*n*=125)						
	
Quality of life tools	Baseline Average (median)±sd	Week 4 visit Average (median)±sd	Δ	p	Week 16 visit Average (median)±sd	Δ	p
MOS-HIV							
Physical component	81.28 (83.14) 9.80	84.84 (86.34) 10.23	3.56%	0.007	86.82 (87.16) 16.98	5.54%	0.003
Mental component	71.31 (75.10) 13.47	76.68 (78.37) 13.61	5.37%	0.001	77.27 (78.88) 10.14	5.96%	<0.001
EQ-5D							
EQ-5D Index Score/Value (population-based preference weights)	0.81 (0.80)±0.21	0.89 (1.0)±0.17	0.08	<0.001	0.92 (1.00)±0.14	0.11	<0.001
EUROQoL							
Index Score	73.29 (75.00)±17.93	77.33 (80.00)±17.06	4.04	0.001	80.60 (80.00)±14.22	7.31	<0.001

